# Frequency and genotypic distribution of GB virus C (GBV-C) among Colombian population with Hepatitis B (HBV) or Hepatitis C (HCV) infection

**DOI:** 10.1186/1743-422X-8-345

**Published:** 2011-07-11

**Authors:** Mónica V Alvarado-Mora, Livia Botelho, Anna Nishiya, Raymundo A Neto, Michele S Gomes-Gouvêa, Maria F Gutierrez, Flair J Carrilho, João RR Pinho

**Affiliations:** 1 Laboratory of Gastroenterology and Hepatology, São Paulo Institute of Tropical Medicine and Department of Gastroenterology, School of Medicine, University of São Paulo, São Paulo, Brazil; 2 São Paulo Blood Bank, São Paulo, Brazil; 3 Department of Pathology, School of Medicine, University of São Paulo, São Paulo, Brazil; 4 Laboratory of Virology, Department of Microbiology, Pontificia Javeriana University, Bogotá, Colombia

## Abstract

**Background:**

GB virus C (GBV-C) is an enveloped positive-sense ssRNA virus belonging to the *Flaviviridae *family. Studies on the genetic variability of the GBV-C reveals the existence of six genotypes: genotype 1 predominates in West Africa, genotype 2 in Europe and America, genotype 3 in Asia, genotype 4 in Southwest Asia, genotype 5 in South Africa and genotype 6 in Indonesia. The aim of this study was to determine the frequency and genotypic distribution of GBV-C in the Colombian population.

**Methods:**

Two groups were analyzed: i) 408 Colombian blood donors infected with HCV (n = 250) and HBV (n = 158) from Bogotá and ii) 99 indigenous people with HBV infection from Leticia, Amazonas. A fragment of 344 bp from the 5' untranslated region (5' UTR) was amplified by nested RT PCR. Viral sequences were genotyped by phylogenetic analysis using reference sequences from each genotype obtained from GenBank (n = 160). Bayesian phylogenetic analyses were conducted using Markov chain Monte Carlo (MCMC) approach to obtain the MCC tree using BEAST v.1.5.3.

**Results:**

Among blood donors, from 158 HBsAg positive samples, eight 5.06% (n = 8) were positive for GBV-C and from 250 anti-HCV positive samples, 3.2%(n = 8) were positive for GBV-C. Also, 7.7% (n = 7) GBV-C positive samples were found among indigenous people from Leticia. A phylogenetic analysis revealed the presence of the following GBV-C genotypes among blood donors: 2a (41.6%), 1 (33.3%), 3 (16.6%) and 2b (8.3%). All genotype 1 sequences were found in co-infection with HBV and 4/5 sequences genotype 2a were found in co-infection with HCV. All sequences from indigenous people from Leticia were classified as genotype 3. The presence of GBV-C infection was not correlated with the sex (p = 0.43), age (p = 0.38) or origin (p = 0.17).

**Conclusions:**

It was found a high frequency of GBV-C genotype 1 and 2 in blood donors. The presence of genotype 3 in indigenous population was previously reported from Santa Marta region in Colombia and in native people from Venezuela and Bolivia. This fact may be correlated to the ancient movements of Asian people to South America a long time ago.

## Background

GB virus C/Hepatitis G virus (GBV-C/HGV) is an enveloped, positive-sense, single-strand RNA virus belonging to the family *Flaviviridae *with a genomic size of about 9.3 Kb [1]. It is genomic organization mainly consists of a large open reading frame (ORF) that encodes a single polyprotein precursor in which the structural (E1 and E2) and nonstructural proteins (NS2 to NS5B) are positioned at the N-terminal and C-terminal end, respectively [2]. It was first identified in 1995 in serum from individuals with idiopathic hepatitis [3]. Although it was initially identified as a possible etiological agent of viral hepatitis in humans, and despite its similarity in genome structure with hepatitis C virus (HCV), in contrast to HCV, GBV-C does not appear to be a hepatotrophic virus neither replicates in hepatocytes nor causes acute or chronic hepatitis [4-6].

GBV-C can be transmitted parenterally through blood and derivates transfusion, intravenous drug use, hemodialysis and vertical transmission [7-10]. There is extensive evidence that GBV-C is transmitted by sexual and percutaneous routes and is frequently found in populations at risk for blood-borne or sexually transmitted viruses [5,11]. Male to male sex has been reported as an effective way of transmission [12] and intrafamilial transmission has been determined based on the sequences analysis [13,14]. GBV-C has not been associated with any particular disease despite numerous investigations. Alteration in the host's cellular immune response to HIV seems to be responsible for a protective effect of GBV-C but the exact mechanism to it still have to be defined. In contrast, GBV-C infection does not appear to have any effect on chronic liver disease due to HCV or HBV [12].

GBV-C infection is relatively common and has a worldwide distribution. At least 1 to 4% of healthy blood donors have GBV-C RNA [1,5,7]. Most people clear the virus and develop antibodies to the E2 envelope glycoprotein. Furthermore, infection is common in the normal population with up to 12.9% prevalence among paid blood donors in the United States of America [15], 11% to 14% in West Africa or South Africa [8], and as high as 37% among HIV-infected individuals [16]. GBV-C incidence amongst of patients with HCV infection varies from 11 to 24% [17-19]. Further, little work has been done on coinfection of GBV-C and HBV but no significant effect of this co-infection was reported [12].

The geographical distribution of GBV-C is related to the coevolution of the viruses with human during the migration along the history, suggesting that GBV-C is an ancient virus [20,21]. Genotype 1 is found in West Africa [22], genotype 2 (sub-classified as both 2a and 2b) in the United States and Europe [23], genotype 3 in Asia [24-26], genotype 4 in Myanmar and Vietnam [27], genotype 5 in South Africa [28] and genotype 6 in Indonesia [29]. In South America, the genotypes 1, 2a, 2b and 3 have been reported [30-34]. In Colombia, a high prevalence of GBV-C RNA and presence of genotype 3 were found among Colombian native Indians from Wayuu, Kamsa and Inga ethic groups [34].

The aim of this study was determined the frequency of GBV-C RNA and the GBV-C genotypes circulating in Colombia. This is the first study that characterizes the presence of GBV-C among blood donors in coinfection with HCV and HBV in Colombia.

## Materials and methods

### Study Population

In order to identified the frequency of GBV-C in Colombia, i) 408 samples from Colombian blood donors positive for anti-HCV (n = 250) and positive for HBsAg (n = 158) using third generation ELISA in the blood bank of Cruz Roja Colombiana in Bogota city, Colombia, and ii) 99 samples from indigenous people with HBV infection from Leticia, Amazonas were obtained for this study. These samples were collected between 2003 and 2007 and the presence of HCV RNA and HBV DNA were previously reported by our group [35,36].

The protocol of this study was approved from Ethical Committees from the Pontificia Universidad Javeriana, Bogotá, Colombia and University of São Paulo Medical School, São Paulo, Brazil. All patients have signed an informed consent before joining the study.

### GBV-C RNA Extraction

To avoid false-positive results, rigorous procedures used for nucleic acid amplification diagnostic techniques were followed [37]. HCV-RNA extraction was carried out from 140 ml of serum using QIAamp Viral RNA Kit (QIAGEN, Valencia, CA), following the manufacturer's instructions. The synthesis of the complementary DNA (cDNA) was carried out immediately after RNA extraction.

### Synthesis of the complementary DNA (cDNA)

Reverse transcriptase reaction was performed using the Moloney Murine Leukemia Virus Reverse Transcriptase (MMLV-RT) and random primers. The final volume of the reaction was 60 ml in the following concentrations: 50 mM Tris-HCl (pH 8.3), 75 mM KCl, 3 mM MgCl_2_, 10 mM DTT, 0.5 mM of each dNTP, 450 ng random primers, 30 U RNAse enzyme inhibitor (RNaseOUT™), and 300 U MMLV-RT. Samples were submitted to the following temperature cycles: 70°C for 10 min, 25°C for 15 min, 37°C for 60 min, and 95°C for 15 min in a thermocycler (Eppendorf Mastercycler 1, Eppendorf, Hamburg, Germany).

### Polymerase chain reaction (PCR)

A fragment of 344 bp from 5' untranslated region (5'UTR) was amplified by nested RT PCR [28,38]. Amplification consisted of 40 cycles for first and second round of PCR, with the following incubation times and temperatures: 94°C 30 s, 50°C 30 s and 72°C 30s for the first round and 94°C 30s, 60°C 30 s and 72°C 30s for the second round.

### GBV-C Sequencing

Amplified cDNA was purified using the ChargeSwitch PCR Clean-Up Kit. Sequencing was done in an ABI Prism 3500 Automatic Sequencer (Applied Biosystems, Foster City, CA) using dideoxy nucleoside triphosphates (ddNTPs) containing fluorescent markers (Big Dye1 Terminator v3.1 Cycle Sequencing Ready Reaction Kit-Applied Biosystems).

The consensus sequences were obtained by alignment of both sequenced strands (sense and antisense) using the SEQUENCHER software (Gene Codes Corporation Ann Arbor, Michigan, United States of America).

### Phylogenetic Analysis

The sequences obtained in this work were genotyped by phylogenetic reconstructions using reference sequences from each GBV-C genotype obtained from GenBank (n = 160). Sequences were aligned and edited using Clustal X [39] and Se-AL (available at: http://tree.bio.ed.ac.uk/software/seal/) softwares respectively. Bayesian phylogenetic analyses were conducted using the Markov Chain Monte Carlo (MCMC) simulation implemented in BEAST v.1.5.3 [40]. The dataset was analyzed under relaxed uncorrelated lognormal and relaxed uncorrelated exponential molecular clock using the best model of nucleotide substitution (GTR+G+I) chosen by MODELTEST [41] and 20 million generations were sufficient to obtain the convergence of parameters. The molecular clock that best fitted the data was chosen by Bayes factor (BF) comparison. The maximum clade credibility (MCC) tree was obtained from summarizing the 20,000 substitution trees after excluding 10% of burn-in using Tree Annotator v.1.5.3 [40]. Phylogenetic trees were visualized in FigTree v.1.2.2 (available at: http://tree.bio.ed.ac.uk/software/figtree/).

### Statistical analyses

Statistical analyses were performed using Minitab Software v. 15. The χ^2 ^test for linear trend (α = 0.05) was used to examine the variations in the presence of GBV-C RNA adjusted for age group, sex and co-infection group (Anti-HCV or HBsAg).

## Results

### Detection of GBV-C RNA

Among Colombian blood donors, from 158 HBsAg positive samples, 5.06% (n = 8) were positive for GBV-C RNA and from 250 anti-HCV positive samples, 3.2% (n = 8) were positive for GBV-C. Among 99 indigenous people from Leticia (n = 99), 7.7% (n = 7) GBV-C positive samples were found (Table 1).

**Table 1:** Geographical origins and epidemiological data from 23 Colombian GBV-C infected patients

Patient	Origin	Clinical Status	Sex	Age	HBV	HCV	Genotype
1035	Bogotá	Blood donor	F	26	Positive	Negative	1
1087	Bogotá	Blood donor	F	28	Positive	Negative	1
1123	Bogotá	Blood donor	M	50	Positive	Negative	2a
1183	Bogotá	Blood donor	F	30	Positive	Negative	3
1196	Bogotá	Blood donor	F	30	Positive	Negative	1
1206	Bogotá	Blood donor	F	22	Positive	Negative	1
1075	Bogotá	Blood donor	M	41	Positive	Negative	No sequence
1174	Bogotá	Blood donor	F	23	Positive	Negative	No sequence
2002	Bogotá	Blood donor	F	30	Negative	Positive	2a
2117	Bogotá	Blood donor	M	63	Negative	Positive	2a
2136	Bogotá	Blood donor	M	63	Negative	Positive	2a
2238	Bogotá	Blood donor	M	59	Negative	Positive	2b
2221	Bogotá	Blood donor	F	21	Negative	Positive	2a
2033	Bogotá	Blood donor	M	37	Negative	Positive	3
2244	Bogotá	Blood donor	F	33	Negative	Positive	No sequence
2259	Bogotá	Blood donor	M	23	Negative	Positive	No sequence
02	Leticia	Native Ticuna	F	67	Positive	Negative	3
95	Leticia	Native Ticuna	F	54	Positive	Negative	No sequence
98	Leticia	Native Yaua	M	51	Positive	Positive	3
113	Leticia	Native Yaua	F	23	Positive	Negative	3
120	Leticia	Native Ticuna	M	43	Positive	Negative	3
148	Leticia	Native Ticuna	F	23	Positive	Negative	3
174	Leticia	Native Ticuna	F	26	Positive	Negative	3

### Phylogenetic Analysis

**Figure 1:**
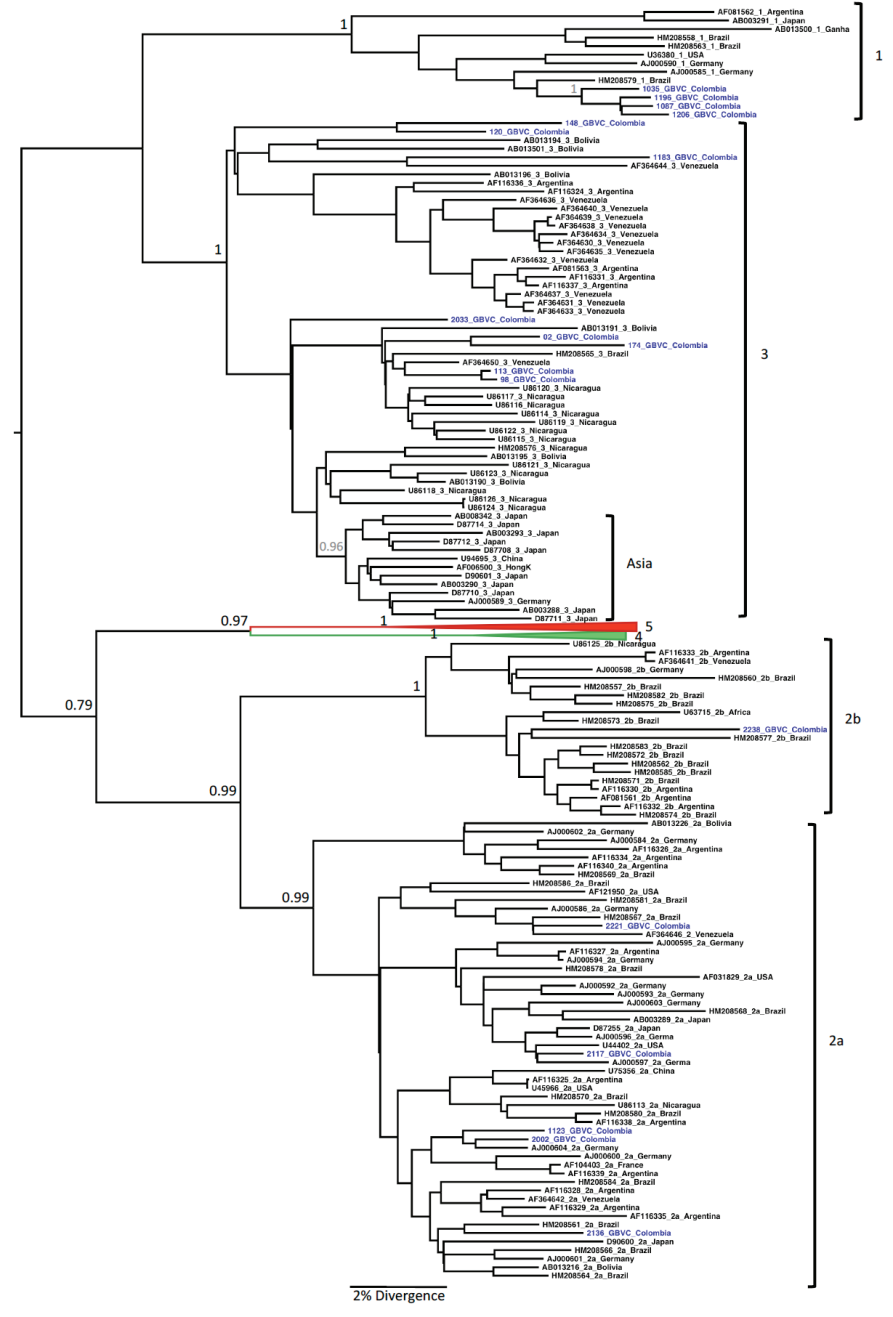
**The maximum clade credibility (MCC) tree was estimated by Bayesian analysis of 160 sequences with 344 bp of GB virus C strains**. The posterior probabilities of the key nodes are depicted above the respective nodes. Samples obtained from Colombian blood donors (n = 12) and Amerindian population (n = 6) were analyzed together with other worldwide strains. Genotype 4 and genotype 5 branches were collapsed.

Eighteen out of 23 sequences with good quality were used for phylogenetic analysis. The phylogenetic tree constructed with the GBV-C sequences (n = 160) is shown in Figure 1. This analysis revealed the presence of the following GBV-C genotypes among blood donors: 2a (41.6%), 1 (33.3%), 3 (16.6%) and 2b (8.3%). All genotype 1 sequences were found in co-infection with HBV and 4/5 sequences genotype 2a were found in co-infection with HCV. All sequences from indigenous people from Leticia were classified as genotype 3. The presence of GBV-C infection among blood donors group and indigenous people was not correlated with sex (p = 0.43), age (p = 0.38) or origin of the samples (p = 0.17). The Colombian GBV-C sequences were deposited in the GenBank database under accession numbers JF832366 to JF832383.

## Discussion

In this study, we determined the frequency and genotypic characteristics of GBV-C virus in Colombia. Among Colombian blood donors, from 158 HBsAg positive samples, eight (5.06%) were positive for GBV-C and from 250 anti-HCV positive samples, eight (3.2%) were positive for GBV-C. This is the first report showing the frequency of GBV-C in HBV and HCV positive blood donors in Colombia.

There are few studies that reported GBV-C frequency in blood donors populations. In Salvador and in Rio de Janeiro, Brazil it was reported 10% of frequency of GBV-C among blood donors [42,43]. Also, in São Paulo, Brazil it was reported a high prevalence among blood donors with normal and elevated ALT levels: 5.2% (5/95) and 6.5% (5/76), respectively [14]. Furthermore, the prevalence of GBV-C was 9.7% among 545 blood donors in São Paulo [44] and 8.3% in 1.039 healthy individuals [45].

In Iranian volunteer blood donors the prevalence of GBV-C was around 1% [46]. A study of prevalence of GBV-C among northeastern Thai blood donors carrying HBsAg and anti-HCV revealed a higher frequency of GBV-C RNA (10% and 11%, respectively) in the co-infected when compared with the controls [47]. In United States, GBV-C prevalence in blood donors was reported ranging from 0.8% to 12.9% [48,49]. In Turkey the prevalence of GBV-C was 14% in hemodialysis patients and 5% in blood donors [50]. Also, in Thailand the GBV-C RNA positivity among blood donors was 4.8% [51]. In France, among 306 HCV RNA-positive donors, 19.3% were GBV-C RNA positive [52]. In Egypt, El-Zayadi et al., [53] found 12.2% of GBV-C prevalence among blood donors and GBV-C coinfection in HBV and HCV infected patients were 7.6 and 64.9%, respectively. GBV-C infection is generally more common in groups with risk factors for percutaneous and sexual transmission of infectious agents [54].

The presence of GBV-C infection in our study was not correlated with sex (p = 0.43), age (p = 0.38) or origin (p = 0.17). These results are correlated with the previous study performed among three different Indian groups from Colombia (Wayuu, Inga and Kamsa) where no significant differences were found [34].

A phylogenetic analysis revealed the presence of GBV-C genotypes 2a, 1, 3 and 2b among Colombian blood donors. There are few studies that reported GBV-C genotypes among blood donor populations in the world. In Bolivia, among blood donors, it was found that the major genotype was genotype 3 followed by genotype 2 [32]. In Shanghai, China, genotype 3 was the most prevalent [55]. In Martinique, Césaire et al., [56], reported genotypes 2a, 1 and 2b among blood donors.

Furthermore, all genotype 1 sequences from Colombian blood donors were found in co-infection with HBV and 4/5 sequences from blood donors genotype 2a were found in co-infection with HCV, it was not found a significant association between the presence of HBV or HCV co-infection and GBV-C genotype (p = 0.431). These results are similar to other studies where no significant differences of HBV, HCV and GBV-C infection rates were found [57].

Phylogenetic analysis showed that genotype 3 is the most common in Leticia, Amazon region of Colombia. The finding of an Asian GBV-C genotype in the Americas was first suggested by the analysis of 5'UTR hemophiliac patients from nine locations in Nicaragua [58], Colombian Amerindians [34] and Bolivia [32]. Similarity between Nicaraguan and Asian GBV-C genotype 3 strains indicates that these strains in the region presumably have an Amerindian origin [58].

In Colombia, from 163 native Indians, 6.1% (n = 10) were positive for GBV-C RNA and it was concluded that the incidence of GBV-C infection in native Indians tended to be high compared with the general population [34]. Furthermore, most Colombian native Indians harbored an Asian GBV-C genotype.

In an Amerindian population from Venezuela, a high prevalence of GBV-C genotype 3 was observed, ranging from 5% (9 out of 162) in the West to 25% (14 out of 56) in the south region of the country [33]. Whereas GBV-C genotypes 1, 2 and 3 were presented in Venezuela, genotype 3 (Asian genotype) was found infecting Amerindians and rural population [33].

Together with other studies, our results corroborate the hypothesis that GBV-C is an old virus [21], having been probably introduced in the America continent with the fist men coming thought the Bering Strait [32-34,58]. It is generally believed that Colombian native Indians migrated from Asia to Colombia approximately 12,000 years ago and were isolated from other people for religious reasons [59]. On the other hand, since HTLV-1 or HTLV-2 may have been brought from Asia to Colombia together with the first human migrants [60], GBV-C/HGV apparently followed the same route [34]. These results are correlated with the evolutionary analysis of GBV-C performed by Suzuki et al., [61], that assumed that this virus diverged 100.000 years ago. However, further analysis needs to be performer to test this hypothesis. Actually, more sequences of GBV-C genotype 3 need to be reported with sampling date, allowing to estimate a specific substitution rate for this genotype that is needed for a more detailed phylogeographic analysis.

## Conclusions

In conclusion, this is the first study that reported the GBV-C frequency and the distribution of genotypes among Colombian blood donors. The result presented indicated the circulation of the genotype 3 among Amerindian population in Colombia and blood donors.

## Competing interests

The authors declare that they have no competing interests.

## Authors' contributions

MVAM conducted the phylogenetic and evolutionary analysis, drafted the manuscript and participated in its design and coordination. LB, AN and MSGG participated in the PCR amplification and sequencing process. RAN conducts the statistical analysis. FJC and MFG participated in the design of the study. JRRP participated in the elaboration of the manuscript. All authors read and approved the final manuscript.
